# Immunology of Physical Exercise: Is *Equus caballus* an Appropriate Animal Model for Human Athletes?

**DOI:** 10.3390/ijms25105210

**Published:** 2024-05-10

**Authors:** Olga Witkowska-Piłaszewicz, Katarzyna Malin, Izabela Dąbrowska, Jowita Grzędzicka, Piotr Ostaszewski, Craig Carter

**Affiliations:** 1 Department of Large Animals Diseases and Clinic, Institute of Veterinary Medicine, Warsaw University of Life Sciences, Nowoursynowska 166, 02-787 Warsaw, Poland; 2 Department of Population Health and Reproduction, School of Veterinary Medicine, University of California, Davis, CA 95616, USA; 3 Department of Physiological Sciences, Faculty of Veterinary Medicine, Warsaw University of Life Sciences, Nowoursynowska 159, 02-776 Warsaw, Poland; 4 Veterinary Diagnostic Laboratory, University of Kentucky, Lexington, KY 40506, USA; craig.carter@uky.edu

**Keywords:** sports medicine, exercise immunology, lymphocytes, cytokines, fitness, animal model

## Abstract

Domestic horses routinely participate in vigorous and various athletic activities. This enables the horse to serve as a model for studying athletic physiology and immunology in other species, including humans. For instance, as a model of physical efforts, such as endurance rides (long-distance running/aerobic exercise) and races (anaerobic exercise), the horse can be useful in evaluating post-exercise response. Currently, there has been significant interest in finding biomarkers, which characterize the advancement of training and adaptation to physical exercise in the horse. The parallels in cellular responses to physical exercises, such as changes in receptor expression and blood cell activity, improve our understanding of the mechanisms involved in the body’s response to intense physical activity. This study focuses on the changes in levels of the pro- and anti-inflammatory cytokines and cellular response in the context of post-exercise immune response. Both the direction of changes in cytokine levels and cellular responses of the body, such as proliferation and expression of surface markers on lymphocytes, monocytes and neutrophils, show cross-functional similarities. This review reveals that horses are robust research models for studying the immune response to physical exercise in human athletes.

## 1. Introduction

Sports medicine focuses on optimal physical fitness, injury prevention and treatment. Professional human athletes rely on a physician to oversee athletes and their teams regularly, via serial blood panels. Red blood cell count (RBC), iron, vitamin B12 and B11, creatinine kinase (CK), urea, aspartate transaminase (AST), alanine transaminase (ALT), cortisol, testosterone and C-reactive protein (CRP) are some of the blood parameters most commonly used to evaluate human athletes. Currently, a reliable biomarker for measuring fitness or evaluating injury risk does not exist [1]. Thus, the discovery and validation of these biomarkers should be a high priority for the medical and scientific community. Of course, the university institutional review boards (IRBs) make such studies on human subjects often difficult or not feasible.

Comparative medicine and animal modeling can be traced back to the 6th century BCE. Animals utilized as comparative models of pathology and physiology must somewhat match the species they are being compared to, e.g., humans. In recent times, comparative animal models have played an important role in polio vaccine development and insulin isolation. They are a warrant of genetic research [2] with unmatched contribution to modern research. The equine model—of importance in translational medicine—has been documented in regenerative medicine [3,4], joint-cell therapy studies [5] and osteoarthritis research [6]. As an illustration, the pathophysiology of exercise-related injuries of the Achilles tendons in humans has been investigated using equine cell culture and tissue explant models. 

The histological and physiological similarities compensated for the limited access to human tissue or specimens in some cases [7]. The horse model is also used in exercise physiology and pathology studies in humans [6,7,8,9,10,11]. In contrast to humans, horses are distracted by professional work, which can disturb a physical training program through additional stress. In one review, it was postulated that the equine immune system’s response, especially to infectious agents and allergens, can be directly compared to that of humans [12].

## 2. Anti-Inflammatory State vs. Open Window Theory in Humans and Equine Model

Physical exercise, depending on its duration and type (endurance, strength training or racing), stimulates the immune system in various ways in both humans and equids [13,14]. In humans, strenuous, acute exercise can cause immunosuppression, referred to as “the open window theory” or “the elite athlete’s paradox”, and can lead to short-term susceptibility to infection [15]. On the other hand, it may result in immunomodulatory action [16]. Generally, exercise-induced immunosuppression is related to moderate-to-high-intensity exercise (55–75% oxygen capacity) lasting longer than 90 min [15,17]. One possible cause is the change in margination and redistribution of peripheral and central populations of white blood cells (WBC) and decreased leukocyte activation [15]. It has also been linked to an increase in Th2 lymphocytes levels [18] and the ability to produce immunosuppressive cytokines [17]. It has been confirmed that human athletes suffering from upper respiratory tract infections produce about 2.5 times more interleukin 10 (IL-10) and interleukin 4 (IL-4) than healthy athletes [17]. Comparable findings have been reported in equine athletes. Under similar circumstances, equine athletes are susceptible to viral infections, such as influenza [19]. 

Additionally, strenuous exercise is known to predispose equine athletes to lower respiratory tract bacterial infections. Similar strains (ex. *Streptococcus* spp., *Pasteurellaceae*, *Microcorcus* spp., *Bacillus* spp.) are present in both pre- and post-exercise horse bacterial isolate samples, but at much higher concentrations in the latter. Immunosuppression contributes to the incidence of pneumonia and other bacterial and viral infections in racehorses [20,21,22]. However, recent studies are disputing the “open window” concept, shifting toward environmental factors, such as participation in mass events, traveling, changes in time zones and temperature, sleep disorders, fatigue, altered or inadequate diet, dehydration, stress and pollution. Inhalation of cold and dry air, with an increased rate and depth of breathing during exercise, can predispose to opportunistic infections in humans [16]. A closer look at some earlier studies in horses also points to environmental factors as causes of respiratory problems [20,21,22]. 

Other studies suggest that moderate, short-term but regular exercise can be beneficial. Chronic exercise modulates the immune response in both species [10,23]. Physical activity is recommended in concert with the pharmacological treatment of many diseases in humans [24]. In 2007, the American College of Sports Medicine (ACSM) initiated the worldwide Exercise Medicine^®^ program. The aim was to introduce physical activity as a standard in clinical care for humans. In an extensive study involving over 1000 participants, it was shown that aerobic activity at least 5 times a week for 20 min reduced the risk of upper respiratory tract infections by 43% in humans [25]. To confirm the positive effect of exercise, it was documented that regular joggers, on average, took 1.5–2.8 days of sick leave per year compared to 4.4 days in the general USA population [26]. 

Further, it was shown that post-exercise immunomodulation creates an anti-inflammatory state, not a suppression of the immune system [16,27]. Interestingly, exercise was recommended as a helpful tool against COVID-19 [28]. A similar anti-inflammatory state is seen in the horse. During endurance and race training, a decrease in cytokine type 1 production is seen (e.g., IL-1, IL-6) [10,29]. In addition, in well-trained horses, the immune cells have strong anti-inflammatory competence [11]. Recent research suggests that post-exercise immunosuppression might have a positive outcome in horses, creating a state of tolerance to slight muscle damage similar to that seen in humans [30].

## 3. Exercise-Induced Acute Phase Response in Both Species

Physical exercise is one of the most physiologically stressful events in humans and horses. Depending on the intensity and duration, this activity can result in various changes in the athlete’s body. High-intensity exercise can cause tissue inflammation [31]. Similar to infection, exercise initiates a sequence of defense reactions, known as acute phase response (APR). APR is a well-understood mechanism in mammalian species. Its onset is correlated with the duration and intensity of physical exercise [32,33]. APR leads to changes in liver production of several proteins. As a result, the plasma concentrations of some acute phase proteins (APPs), known as positive APPs, increase. Among them are serum amyloid A (SAA), C-reactive protein (CRP), haptoglobin, ceruloplasmin, fibrinogen and alpha-1-acid glycoprotein. There are also other proteins involved. The production of negative APPs decreases during APR. These include albumin, transthyretin, transcortin and retinol-binding protein. 

The pro-inflammatory processes that follow intensive exercise, such as increased expression of pro-inflammatory cytokines, may be essential for the long-term adaptive responses to training. The typical APR occurring in exercise-induced inflammation involves increases in the concentrations of pro-inflammatory cytokines (TNF-α, IL-β and IL-6) and APPs. In humans, CRP has been identified as a major APP, and it is a sensitive but non-specific marker of inflammation [34]. A recent systematic review has been conducted in accordance with the *Preferred Reporting Items for Systematic Reviews and Meta-Analysis* (PRISMA) statement guidelines, showing that CRP increased both after intense and moderate exercise, with peaks as late as 28 h post-exercise in humans [35].

A similar reaction was shown in horses after strenuous endurance exercise and racing; however, there were some differences. In horses, exercise-induced APR is characterized by heightened SAA, a major APP in this species [36]. A minimum of a 10-fold increase in SAA blood concentration was documented in horses completing endurance rides over the longest distances (120 and 160 km), but not shorter (up to 60 km) [37,38], with unchanged concentrations of CRP and haptoglobin [39]. In a more recent study, SAA increase occurred only in inexperienced endurance horses after a 100 km ride [40]. Additionally, in a study performed on 47 elite endurance horses, there was no SAA increase after a 160 km ride. Similar findings with no increase in SAA after exercise were obtained in racehorses [37,41,42]; however, in a more recent study, in an inexperienced group, a slight elevation in SAA occurred after exercise [40], as well as one and two days after the race [43]. 

In humans, several other proteins are affected in response to inflammation, the majority showing increased levels shortly after the inflammatory reaction [44]. Ceruloplasmin (CP) plays a crucial role in the metabolic balance of copper and iron through its oxidase function and exhibits antioxidant activity. CP can reduce the formation of free radicals in exercising human athletes. In endurance horses, exercise did not trigger the changes in CP [39]. In a more recent study, APR was characterized by an 18% increase in the concentration of CP, which was linked to higher average speeds [45].

Another APP, which may be altered by exercise, is haptoglobin (Hp), which represents the most important plasma detoxifier of hemoglobin [46]. Exercise-induced hemolysis, defined as the rupture and destruction of erythrocytes, is reflected by a significant decrease in plasma Hp and a significant increase in plasma concentration of free hemoglobin. In turn, more intense exercise may increase hemolysis, which leads to a significant decrease in Hp levels [47]. Several studies report a significant decrease in Hp after long-distance running events in humans. In one study involving a large group of male and female marathon competitors (n = 90 and n = 20), the Hp plasma level decreased by 1.4- and 1.8-fold, respectively [48]. Moreover, the longer the endurance exercise was taken, the higher decrease in Hp was observed. Participants in two triathlon races, which differed in the length of the distances, expressed a 32% vs. 20% decrease in Hp serum level. Thus, it was concluded that the Hp level decrease is specific for runners and is dependent on the training intensity [47,49]. However, one study showed that a significant Hp serum level decrease is also true for sprinters [50].

A less discernible but similar tendency of Hp change after exercise was observed in endurance horses. An 11% decrease in serum Hp was observed in horses after endurance competition. This change was proven to be even greater after a longer distance [45]. The racehorse training session also resulted in decreased Hp serum levels. An over 40% decrease was observed in Standardbred trotters after racing a 1600–2500 m distance [42]. Similar effects were observed in a study performed by Masini et al. (decrease of 17%) [51] and Cywińska et al. [52]. Thus, the Hp changes in horses are similar to those observed in human athletes. 

## 4. Cytokine Response

Physical exercise and its physiological consequences require an integrated cytokine response, as they are the major signaling molecules regulating immune pathways. Since exercise promotes trauma in muscles (metabolic depletion and tissue damage), this triggers a pro-inflammatory response. Simultaneously, anti-inflammatory processes are promoted to counteract the homeostatic disturbance [53]. Many studies refer to the cytokine profile as a useful biomarker for monitoring an athlete’s performance, recovery in the course of training and overall well-being [54,55]. In both humans and horses, the source of immune response to training is the muscles, which secrete cytokines [56,57]. In both species, exercise triggers a different response in the muscles than in the blood. This can be interpreted as changes in the muscles being a form of acute adaptation and those in the blood as a direct but delayed response to muscle damage and inflammation caused by physical activity. 

In horses and in humans, some parameters (e.g., IL-6) rise mostly in the muscle, while others are blood-specific (e.g., IL-1), or blood is the secondary source [54,58]. Cytokine profiles, particularly the contribution of pro- and anti-inflammatory cytokines, correlate with the performance and progress in adaptation to training [59,60,61]. Additionally, the cytokine response to training may vary depending on factors such as the type, intensity and duration of exercise, individual differences and other factors [53,61,62].

The main pro-inflammatory cytokine involved in exercise-induced immune response in horses and humans is IL-6 [63,64]. The cytokine is a conspicuous marker of APR, stimulated by the depletion of glycogen in the working muscles and/or microdamage of muscle fibers. Thus, it is a pivotal player in muscle metabolism [57,58,65]. The upregulation of IL-6 has been proven to be in direct proportion to the intensity and duration of the effort [66,67]. Research shows that IL-6 increases up to 100-fold post-exercise, which is especially observable after endurance exercise lasting longer than several hours [63,68,69]. Dramatic changes have been reported. Athletes participating in the ultra-long-distance race experienced an 8000-fold increase in IL-6 plasma concentration [70]. 

Data concerning horses are not as extensive as those on humans; however, some provide sufficient evidence of an increase in plasma IL-6 concentration in endurance horses [56,63]. However, some studies report no change in post-exercise IL-6 concentration. Liburt et al. noticed no changes in this cytokine level in horse blood after the incremental exercise test (GXT) [54]. Similarly, exhaustive studies on humans are in line with this finding [71,72]. Caution is required when drawing conclusions from experiments regarding IL-6, since its functioning is two-fold and dependent on individual traits, as well as athletes’ adaptation to training. It can be considered a pro- or anti-inflammatory cytokine depending on the form of signaling (classical or trans-signaling) [11,73].

Other pro-inflammatory cytokines have also been proven to fluctuate in response to training, such as IL-1β, IL-4, IL-8 and TNF-α. Regarding IL-1β concentration after exercise, research reports significant upregulation immediately after exercise, with some studies even indicating an increase persisting for several hours or days [74,75]. However, other studies have shown no changes in this cytokine’s concentration after both moderate and intensive exercise, measured as the mRNA expression level as well as the concentration of protein in blood plasma [72,76,77]. Another pro-inflammatory cytokine sharing many pleiotropic functions with IL-1 is TNF-α. For example, they are both secreted by monocytes/macrophages at the onset of an inflammatory process and are capable of IL-6 secretion stimulation [78]. 

In human athletes, TNF-α and IL-1 are known to increase after prolonged, exhausting exercise, but no changes are seen in response to moderate exercise [79]. The horse appears to exhibit a similar response, as reported by several studies [11,54,80]. Furthermore, it has been demonstrated that IL-1β and TNF-α may play a role in the process of the horse’s adaptation to endurance as well as race exercise. In untrained racehorses, elevated PBMC production of these cytokines was observed after training, whereas the PBMCs of a well-trained group of racehorses maintained cytokine levels at a consistent level [11]. 

The transient increase in local pro-inflammatory cytokine production following injury is crucial for tissue regeneration. IFN-γ plays an immunomodulatory role and is a factor in myogenesis inhibition after an injury [81]. In general, in response to acute exercise in humans, IFN-γ tends to be upregulated, similarly to IL-6, IL-1β and TNF-α [82]. This phenomenon might be one of the causes of IL-1β and TNF-α upregulation, since the augmentation in the production of this cytokine has been proven as one of IFN-γ functions [83]. Liburt et al., in their incremental exercise test, showed significant upregulation of this cytokine in horses as well. This was the case for plasma and muscle samples [54]. The transcript level of this cytokine is also increased by training in racehorses [12]. Additionally, IFN-γ might be another pro-inflammatory cytokine marker, which diminishes in the course of adaptation to training in horses [11]. The balanced immune response to exercise is guaranteed by the simultaneous secretion of anti-inflammatory cytokines, which alleviate APR’s consequences, such as excessive and persistent inflammation. 

The most active anti-inflammatory cytokine is IL-10. Exercise results in elevated levels of this cytokine in human athletes [82,84,85]. The same dependency was also proved for equine athletes [63,80]. This increase concerns both the transcript and protein blood level. An interesting study of post-exercise cytokine secretion over time has revealed that peaks of IL-10 and IL-6 occur at nearly the same time, dramatically rising immediately after exercise and gradually decreasing over time. This suggests that IL-10 is responsible for the counter-reaction to the pro-inflammatory action of the IL-6 protein [86]. Indeed, it was confirmed that IL-6 infusion resulted in increased IL-10 response compared to saline infusion [87]. Moreover, the IL-10 level in response to exercise might be a good indicator of both species’ adaptation to training. In human master sprinters and endurance runners, Rosa et al. noticed significantly increased IL-10 concentration after exercise in comparison to control participants [88]. Witkowska-Piłaszewicz et al. also showed a better post-exercise anti-inflammatory state in a well-trained racehorse group in comparison to an untrained group [11].

Similar properties of pro-inflammatory cytokine attenuation (in particular TNF-α and IL-1) were confirmed for IL-4 [89,90]. Moreover, this protein participates in myofiber regeneration, being secreted by eosinophils recruited to the injury site [91] and diminishing ROS production [92]. Increased expression of IL-4 was found following acute aerobic training in humans [82]. However, many studies report no changes in the plasma and mRNA amounts of IL-4 in response to different types of activity [72,76,93]. Considering the relatively low basal concentration of this cytokine and the low magnitude of change (e.g., in comparison to IL-6 or IL-10), the method of detection might be insufficient to find significant changes. Cywińska et al. faced this problem while studying endurance horses [63]. Witkowska-Piłaszewicz et al. found no changes in IL-4 levels in plasma serum throughout the endurance training season of Arabian horses [10]. Interestingly, a racehorse study reported upregulated gene transcription of IL-4 [94]. The following work by Witkowska-Piłaszewicz et al. concerning racehorses proved an increase in IL-4 production, but only in the well-trained group. This interleukin may be considered a marker of horse adaptation to training [11]. This same evidence of fine-tuning of the anti-inflammatory state during regular training through IL-4 secretion was confirmed in human athletes [95,96].

Together with IL-4, IL-13 serves as a regulatory protein, which promotes myogenesis. Moreover, in a very recent study published in the prestigious journal *Science*, it was confirmed that IL-13 promotes the metabolic shift to reduce glycogen usage and improve endurance capacity by enhancing mitochondrial respiration and fatty acid utilization by the working muscle [97]. Although IL-13 is rarely referenced in exercise studies, similarly to IL-4, it has been identified as a significant factor in human muscle adaptation to training [96]. In horses, there is still a lack of extensive studies encompassing this cytokine’s contribution to exercise physiology. However, some of them confirm its major role in the performance improvement process and post-exercise response [61,98]. The comprehensive study by Plisak et al. demonstrated the variability of this anti-inflammatory cytokine’s response to different types of horse training (aerobic and anaerobic) [61].

There is growing interest in cytokine use as a marker of athlete performance and well-being. A few more proteins have also been proposed to contribute to the immune response to exercise—among others, a novel anti-inflammatory IL-1Ra and pro-inflammatory IL-17, IL-18, IL-12 and IL-15 [11,57,64,99]. In a study evaluating over a thousand horses, three potential blood markers for early detection of animals at risk of injury were selected (IGF-1, IL-1RN and MMP2) [100]. However, the main limitation in the research connected with cytokine reaction is the paradigm of investigation, which needs to be extrapolated, including the source of the sample, time of collection and exercise design. The source of the cytokine concentration measurement might be the cause of variability in the detected ranges (in the muscle, the changes are more pronounced than in blood, but this is an invasive study). Furthermore, drawing conclusions based on mRNA data might be misleading, as effector protein expression is highly affected by transcriptional and post-transcriptional regulation [101]. Considering this, more reliable information regarding an athlete’s condition might be obtained via the evaluation of protein concentration [10].

## 5. Cellular Response

Due to their physiological similarities to humans, equine models can be valuable tools for studying cellular responses in various human diseases, especially connected with immune cell reactions. Human and equine immune cells share several characteristics in terms of cell surface markers and receptor expression. White blood cell (WBC) population shifts are observed in response to these changes. Initially, increased margination and bone marrow WBC mobilization take place; natural killer cells (NK), T-lymphocytes, macrophages and neutrophils are responsive via β2-adrenergic receptors exclusively in humans, and by 90% in horses, which is a unique similarity [102,103]. This stimulation drives the immediate exercise-induced leukocytosis [82]. Notably, in human studies, all major subpopulations of leukocytes tend to increase in number during exercise, most likely as a result of hemodynamic shear stress and/or catecholamine activation of beta2-adrenergic leukocyte receptors, which are connected to differential expression of beta-2 adrenergic receptors on lymphocytes: NK cells > CD8+ T cells > B cells > CD4+ T cells, including regulatory T cells [16,104]. Lymphocytosis—defined as an increase in the number of T cells and, to a lesser extent, B cells during and immediately following exercise—appears to be proportional to the intensity and duration of the exercise, which is also similar in horses [11,105,106]. In human research, exercise at 80% vs. 50% VO2max caused lymphocytosis immediately after exercise, followed by lymphocytopenia from 1 to 3.5 h and a decrease in the proliferative response of Con A-stimulated lymphocytes (per CD3+ cell) [107]. It should be noted that there is a significant decrease in the number of circulating lymphocytes during acute physical training lasting more than 1 h, regardless of exercise intensity, and throughout the recovery phase after exercise [108,109,110]. The lymphopenia seen in post-exercise recovery is caused by the selective migration of lymphocyte subtypes with strong effector functions, such as T cells, to the peripheral sites of potential antigen contact, such as the lungs and intestines [109]. Additionally, it was found that exercise reduces the fractions of CD3+, CD4+ and CD8+ cells in the total lymphocyte count while increasing the fraction of CD16+ NK cells [111]. 

Limited research has been conducted on the immune cell response in equine athletes, particularly concerning comparative exercise physiology in humans. In the available data, the level of lymphocyte proliferation in racehorses depends on the training level [11,112]. Intense exercise inhibits the proliferation of CD4+ and CD8+ cells in untrained horses, as they tend to show a reduced response to mitogens and antigen-specific stimulation during intense exercise [19]. In contrast, increased physical activity is correlated with increased proliferative activity, similar to human studies [104,113,114,115]. As with humans, long-duration equine exercise may decrease the number of CD4+ cells [116,117], which also depends on the fitness level of the athlete [11] and is parallel with an increase in CD8+ cells in the blood [106]. In more recent studies, it was postulated that this phenomenon is related to CD4+ tissue depletion [16]. In human studies, it is thought that the proportion of B cells increases more than that of T cells [118], while the proportions of CD3+ and CD4+ cells decrease [119]. Unfortunately, there is a paucity of such studies in horses. 

The identification of mature regulatory T cells in humans relies on the expression of CD4+ CD25+. The absence of horse-specific CD25 antibodies and the relatively low homology of the equine CD25 gene have led to the recognition of CD4+ FoxP3+ cells as regulatory T cells, or Tregs [120]. FoxP3 expressions on CD4+ lymphocytes are positively associated with their control and suppressive function in both humans and equines [121]. Endurance exercise in humans was shown to reduce the percentage of Treg lymphocytes while increasing FoxP3 expression in CD4+ CD25+ cells [122]. This was true in trained marathon runners, who had significantly fewer helper 1 and Treg cells and significantly more helper Th2, CD4+ IL10+ and TGFβ+ T cells [123]. This phenomenon is attributed to the exercise’s acute and intense nature. 

An increase in CD4+ FoxP3 and CD8+ FoxP3 levels was observed only in trained racehorses, whereas such studies in endurance horses have not yet been performed [11]. Some differences may exist between horses and humans. A study aimed at identifying regulatory T-cell subpopulations in equine blood lymphocytes discovered that only a small proportion of CD8+ lymphocytes (0.5%) expressed FOXP3, accounting for less than 15% of total FOXP3+ cells [124]. However, a higher percentage of CD8+ FoxP3+ cells was confirmed in horses’ blood in a more recent study [11]. 

NK cells are the immune cells most sensitive to acute exercise [88]. Human studies have demonstrated that NK cells are divided into two distinct subsets based on the intensity of CD56 expression. CD56 (light) and CD56 (dark) cells have distinct phenotypic and functional characteristics. CD56 (dark) NK cells are cytolytic, whereas CD56 (light) NK cells are immunoregulatory principally through cytokine production, with CD56 (dark) cells being more sensitive to intense exercise. It has been found that the post-exercise recovery period increases the ratio of CD56 (light) cells to CD56 (dark) cells [125]. Further research has revealed that acute physical exercise has a strong influence on the absolute and relative NK cell counts in peripheral blood. Depending on the nature of the exercise regimen, a reduction in NK cell numbers has been observed after at least 15–30 min of rest, with this reduction lasting for more than 24 h [126]. 

Regardless, numerous studies have shown that intensive training can increase the number of NK cells, particularly in the baseline [126,127,128,129,130]. Furthermore, moderate-intensity exercise has been shown to increase NK cell cytotoxicity in healthy adults [128]. Further, acute exercise was shown to provoke epigenetic modifications in NK cells, with potential benefits for NK cell function [129]. This finding is complemented by evidence suggesting that acute exercise may stimulate NK cell cytotoxicity through intracellular signaling, e.g., by mediating an increase in perforin levels inside these cells [127]. Importantly, increased NK cell counts and a higher NKCA have been observed in athletes compared to non-athletes [131]. Despite the wealth of research on NK cells in humans, there are few studies investigating their activity during equine exercise [116,132]. Interestingly, horses are reactive to NK-5C6 mAb, implying a similar morphology and physiology to human NK cells. Taken together, these findings highlight the importance of investigating the role of NK cells in exercise in both humans and animals, with potential implications for promoting immune function and overall health [132,133]. Only one study revealed that single bouts of moderate-to-vigorous exercise are associated with an initial increase in NK or LAK cell function in horses [134].

Monocytes are yet another cell population, which play a significant role in the immune response to exercise training. Within the human blood monocyte population, three distinct subtypes are distinguished based on cell surface markers, including classical monocytes (CD14++CD16), non-classical monocytes (CD14+CD16++) and intermediate monocytes (CD14+CD16+) [135]. Each subtype of blood monocyte has a specific role in the immune system, with classical and intermediate monocytes typically exhibiting pro-inflammatory properties and non-classical monocytes being associated with anti-inflammatory actions and mirrored by tissue-resident macrophages (M1 and M2 type cells) [136]. Due to a lack of species-specific monoclonal antibodies, research on the effect of exercise on equine monocyte function has been limited [137,138]. It is hypothesized that CD14-MHCII+ monocytes in horses are thought to be equivalent to human non-classical monocytes, whereas CD14+MHCII+ monocytes are intermediate, and CD14+MHCII- monocytes are classical [11,139]. 

In humans, moderate-to-vigorous exercise increases the percentage of classical monocytes as well as intermediate monocytes [140]. In one study, 1 min of anaerobic exercise increased the number of CD14+CD16+ monocytes 3.5-fold [141]. However, regular physical training induced the lowering of inflammatory (CD14+CD16+) monocytes’ levels [142]. In equine athletes, the findings are similar; however, the existing data are limited. It was found that experienced racehorses showed an increase in CD14-MHCII+ cells after exercise, whereas CD14+MHCII- cells remained unaffected [11]. Untrained horses, on the other hand, showed an increase in CD14+MHCII- cells and a decrease in CD14-MHCII+ monocytes after exercise. 

The proportion of cells with anti-inflammatory properties increases during conditioning, which implies that horse training level may have a significant impact on monocyte counts [11]. Direct comparisons are still fraught with uncertainty due to species differences and the inability to identify the same markers as those used for humans. Additional research and functional studies are required to confirm that regular exercise reduces the number of pro-inflammatory monocytes in horses.

The impact that exercise has on eosinophils in horses and humans has some similarities and differences. In humans, a condition known as “acute eosinopenia” occurs when the number of eosinophils decreases after exercise [143]. The exact mechanisms underlying this phenomenon are not fully understood. This could be due to eosinophils migrating from the blood to other tissues in response to exercise. Cortisol has been shown to suppress eosinophil production and increase migration out of the bloodstream and into tissues. It is important to note that acute eosinopenia after exercise is typically transient, and eosinophil counts usually return to normal levels within a short period. In contrast, one study in horses found a progressive increase in eosinophil count over three months of training in healthy specimens [144]. This suggests that long-term exercise training in horses may have a different effect on eosinophils than in humans.

## 6. Serum Creatine Phosphokinase (CPK)

Serum creatine phosphokinase (CPK) is an enzyme involved in muscle metabolism, and its activity is generally considered a physical stress marker [145]. Leakage of CPK into the plasma has been accepted as an indicator of muscle fiber damage [146]. In endurance horses, CPK and aspartate aminotransferase (AST) are commonly used as indicators of any kind of muscle fatigue or damage. These parameters likewise increase after training and competition, whereas SAA increases only after competition [36]. In humans, the CPK results are variable, as approximately half of the studies demonstrated no alterations immediately after intense or moderate exercise, while the remaining half showed an increase in CPK levels [35]. CPK was the only marker exhibiting a higher increase in moderate exercise compared to intense exercise. Moreover, muscle damage, as evidenced by CPK activity, was not accompanied by parallel markers, namely cytokines and CRP [35]. In a cohort study performed on a large population (5969 men and 6827 women), CPK and CRP were inversely related [147]. Data on the increase in CPK activity as a marker of muscle damage during training are ambiguous. The extent of the results depends largely on the type of exercise, and a significant increase in the activity of this enzyme is not always observed [79,148]. This supports the hypothesis that CPK may have an anti-inflammatory effect in humans, but the direction of the relationship and clinical implications are yet to be investigated in humans as well as in horses. 

## 7. Equine Model in Other Exercise-Related Studies

The equine model has proven to be an invaluable resource for researchers from a variety of scientific disciplines. It has been used to study various aspects of the immune response, including the role of regulatory lymphocytes in immune regulation [12]. Cellular and humoral defense is provided for both species and pro-inflammatory cytokine expression with influenza morbidity. This is associated with specific and similar clinical symptoms. Thus, the same approaches for horse and human vaccine development are currently applied in research [149]. Moreover, in the context of viral diseases, the equine model can explore the advantageous immune mechanism and general host–virus interactions. 

The most common infectious pathogens are structurally and genetically related to those, which infect humans. For example, the equine infectious anemia virus (EIAV) is valuable for studying the human immunodeficiency virus (HIV). An interesting mechanism of EIAV replication control in ponies has been observed as a consequence of transient immunosuppression, which affects further effective humoral response [150]. Understanding this novel immune mechanism could be valuable in the development of a vaccine against HIV [151]. Since the horse provides a comparative research model for sports physiology, the above examples might position this model as essential in research on major sport-related infectious diseases [152,153,154]. Furthermore, the horse model has been used in the development of regenerative methods [3,4]. 

While progress has been made recently on regenerative medicine methods, they are still considered as emerging. Although a few successful regenerative methods have been achieved for horses, pre-clinical data are needed in order to derive human therapy. The equine model provides significant new knowledge in the context of the safety and efficacy of cell-based therapy [155,156]. Furthermore, the physiological and musculoskeletal resemblance and the domestic nature of a horse make this animal a promising candidate for rehabilitation programs’ assessment and post-surgical capacity of weight loaded on joints. 

The equine model has proven particularly useful in the research of joint-cell therapy. Furthermore, the horse has provided unique approaches for researchers to develop new treatments and therapies for osteoarthritis—a serious joint disease in adult human patients [5,6]. In comparison with alternate laboratory animal models for osteoarthritis, though less expensive and easier to handle, horses provide unique strengths for this kind of research. Structural and functional anatomy of synovial joints, cartilage thickness, subchondral bone characteristics and the size of carpal and metacarpal joints are the most important advantageous objectives for equine model utility in osteoarthritis [157,158]. 

Horse athletes are ideal for the investigation of naturally occurring and post-traumatic osteoarthritis, especially since over 60% of their body weight bearing relies on joints [6]. It is worth noting that similar imaging procedures and diagnostic modalities are used for horses and humans. Additionally, the relative size of the horse offers comparably non-invasive arthroscopy and harvests a large volume of synovial fluids [159,160,161]. 

It is important to note that human and horse tendons are alike in structure, composition and biomechanics. For example, common exercise-induced Achilles injuries are the aim of interest in therapeutic research. Important age-related pathophysiology similarities with humans have been noticed in horse tendon injuries [162]. Moreover, tendon disease with the etiology of a particular polymorphism within the extracellular matrix proteins has been proven for both species [163,164,165]. Furthermore, due to the scarcity of tissue specimens in the medical field, equine cell culture and tissue explant models have been used to study the pathophysiology of exercise-related injuries, such as Achilles tendon injuries [7]. The horse model has been widely used in the fields of exercise physiology and pathology, in addition to its utility in specific areas of study. As such, it has contributed significantly to our understanding of the human response to exercise and the pathophysiology of various exercise-related injuries [6,7,10,11]. Overall, the equine model is a valuable resource for researchers from a variety of fields, and it is likely to remain an important resource for advancing scientific understanding and developing new therapies and treatments.

Although the equine model is attractive, some important considerations must be recognized. The acquisition, feeding, humane concerns, sheltering and medical care of horses will add significantly to the cost of any study. Further, the market for reagents (e.g., antibodies) is currently insufficient to support some studies utilizing the equine model. At the molecular level, equine genomic functional and tissue-specific annotation is still not a robust tool, which makes it challenging to extrapolate transcriptomic findings [166,167]. The relatively long timeframe to maturity and ethical issues related to the horse model represent additional obstacles. Finally, depending on the investigation methods, the sheer size of a horse can also be considered a limiting factor. 

All discussed similarities and differences in both species have been summarized in Table 1. 

## 8. Conclusions

Despite drawbacks, the equine model provides many strengths for comparative studies of physiologically and immunologically related health issues. The equine model seems almost ideal for the investigation of immunological responses to physical training because of horses’ athletic abilities and physiological similarities to humans. The cellular reaction, cytokine profile and APP changes during exercise are similar in both species.

## Figures and Tables

**Table 1:** Similarities and differences between humans and horse athletes.

Subject Studied	Findings in Humans		Findings in Equines
**Hormonal Response**			
Catecholamines	Leucocyte count increases proportionally to catecholamine levels during exercise in the form of pseudoleukocytosis—a release of white blood cells from spleen, liver, bone marrow and lymph nodes into the bloodstream [118,168,169]		Exercise provokes increases in plasma concentrations of adrenaline and noradrenaline [105] Causes pseudoleukocytosis [170]
Cortisol	Rises in direct proportion to the intensity and duration of exercise, which exceed a certain threshold [171,172,173]		Rises in response to exercise, causing temporary immunosuppression [162]; on the other hand, the increase is only two-fold [174]
**Humoral Immunity**			
General Findings			
IFN-γ	Increase in moderate exercise [175] Upregulated in response to acute exercise [82]		Increase in transcription when measured immediately after exercise [29] Decrease in the course of horse adaptation to endurance training [11]
Interleukins	IL-1 β, IL-6, IL-8, IL-10 may rise in endurance exercise [62,176] IL-1β may decrease in endurance exercise [176,177] IL-1 is upregulated immediately after exercise and a few days after [74,75] No changes in IL-1 transcript level [72,77] Over 100× IL-1ra and IL-6 increase following endurance activity [76] Increase in IL-6 [77] sourced from the muscle tissue [178] IL-6 increases the most in fastest runners [176] Il-6 increases dramatically after long-lasting exercise [68,70] IL-10 is elevated after exercise [82,84] IL-10 concentration is significantly increased in the blood of experienced runners in comparison to inexperienced runners [88] Increased expression of IL-4 after acute aerobic training in humans [82] IL-4 mRNA amount does not change in response to different types of activity [72,76,93] IL-4 secretion increases along with regular training [95,96] IL-13 secretion increases along with muscle adaptation to training [96]		IL-1β increases in both endurance activity [179] and strenuous exercise [29,54] IL-1β, IL-3 gene expression increased at day 90 of training [99] IL-2, IL-4, IL-8 gene expression increased at days 30 and 90 of training [99] IL-1β and IL-6 mRNA expression increased at 2 h following exercise [29] IL-6 mRNA expression increases in the muscle but not in blood [54] IL-6 is upregulated in direct proportion to the intensity and duration of exercise [63] IL-1 mRNA expression increases in blood but not in the muscle [54] IL-10 increases transcript and protein levels in the blood after exercise [63,80] Higher IL-10 response in experienced racehorses in comparison to untrained racehorses [11] Upregulated IL-4 transcription in racehorses after exercise [94] IL-4 increased the production in horses adapted to training in comparison to the untrained group [11] Increase in IL-13 in endurance horses Higher basal IL-13 serum concentration in untrained racehorses in comparison with well-trained racehorses Decreased IL-13 serum concentration after exercise in untrained racehorses but increased concentration in well-trained racehorses [61]
TNF-α	Increase in marathon runners [180] Decrease after bicycle exercise [77] Increase after prolonged, exhausting exercise but no changes seen in response to moderate exercise [79]		Increase in mRNA expression after the first exercise session in untrained horses and an inverse correlation with IL-6 expression [29] mRNA expression increases in both muscle and blood post-exercise [54] Elevated production in horses unadapted to training; no changes in well-adapted horses [11]
**Cell-Mediated Immunity**			
Lymphocytes	General Findings	Human lymphocytes and neutrophils express β2-AR receptor exclusively [109,181] Increase during exercise, decrease after strenuous exercise [118] Relatively higher increase in B cells than T cells [182] Relatively higher increase in T-cytotoxic than in T-helper cells (Crary et al., 1983) [183] Proportions of CD3+ and CD4+ cells decrease; CD19+ cells do not change [119] Type 1 T-cell population decreases but not type 2 [87] Endurance exercise in humans reduces the percentage of Treg while increasing FoxP3 expression in CD4+ CD25+ cells [122]	High compatibility—90% of equine lymphocyte β-adrenergic receptors are β2; exercise induces upregulation [105] Decrease in cell count [105] Intense exercise inhibits the proliferation of CD4+ and CD8+ cells in untrained horses [19] Increase in the levels of CD4+ FoxP3 and CD8+ FoxP3 in trained racehorses [11]
	NK Cells	Increased count [184]—CD16 and CD56 cell counts increase over 2 h; then, the cells are inhibited by prostaglandins released by monocytes post-exercise [119] Increase in cytotoxic activity especially high in endurance training and proportional to the intensity of the workout [184]	Although the NK response to physical activity in horses has not been described in detail, it is known that the equine population is reactive to NK-5C6 mAb (an antibody reactive to human NK cells), CD3-, CD4-, CD8-, and it is cytotoxic to cells, which do not display MHC I, implying similar morphology and physiology [132]
Granulocytes	Neutrophils	Increased cell count and continual increase post-exercise [118], impaired chemotaxis, decreased adherence [185]	Increased cell number [105], decreased chemotaxis after a single bout [186]
	Eosinophils	Acute eosinopenia [143]	One study showed a progressive increase in EOS count over three months of training in healthy horses [94]
Acute Phase Response	CRP increased both after intense and moderate exercise, with peak increases up to 28 h in humans [35]		Increase in serum amyloid A, fibrinogen and iron [20]
